# Silent Propagation of Classical Scrapie Prions in Homozygous K_222_ Transgenic Mice

**DOI:** 10.3201/eid3112.250302

**Published:** 2025-12

**Authors:** Natalia Fernández-Borges, Alba Marín-Moreno, Juan Carlos Espinosa, Sara Canoyra, Olivier Andréoletti, Juan María Torres

**Affiliations:** Centro de Investigación en Sanidad Animal (CISA-INIA-CSIC), Madrid, Spain (N. Fernández-Borges, A. Marín-Moreno, J.C. Espinosa, S. Canoyra, J.M. Torres); Interactions Hôte Agent Pathogène–École Nationale Vétérinaire de Toulouse, Toulouse, France (O. Andréoletti)

**Keywords:** classical scrapie, prions and related diseases, goat, mouse, resistant genotype, silent prion carrier, Spain, Europe

## Abstract

Classical scrapie affects sheep and goats. To control prevalence in sheep, the European Union initiated breeding programs targeting resilient genotypes. Although certain goat polymorphisms, such as Q_222_K, are linked to resistance, specific breeding programs have not been implemented. Hemizygous transgenic mice carrying the goat K_222_ cellular prion protein (PrP) allele (K_222_-Tg516) exhibited resistance to several classical scrapie isolates. We inoculated homozygous K_222_-Tg516 and Q_222_-Tg501 mice with various scrapie isolates. Homozygous K_222_-Tg516 mice reached the end of their lifespan without exhibiting clinical signs; we observed brain proteinase K–resistant PrP accumulation in those mice that was lower than in Q_222_-Tg501 mice. Histologically, K_222_-Tg516 brains lacked prion-related lesions, except for the presence of few isolated scrapie PrP plaques in cases of isolates highly adapted to the K_222_-PrP^C^ environment. Our findings caution against including that polymorphism in breeding programs, because it could lead to emergence of asymptomatic silent prion carriers of classical scrapie among goat populations.

Scrapie is a fatal infectious neurodegenerative disease inherent to sheep and goats that falls within the spectrum of transmissible spongiform encephalopathies (TSEs) or prion diseases. Of note, various mammals, including cattle with bovine spongiform encephalopathy (BSE), mink with transmissible mink encephalopathy, cervids with chronic wasting disease, and humans with Creutzfeldt-Jakob disease, can also succumb to TSEs. The hallmark of those diseases is posttranslational conversion of the host cellular prion protein (PrP), PrP^C^, into a misfolded pathologic isoform causing scrapie, PrP^Sc^, which accumulates within the central nervous system of affected individuals (*1*).

Infection with TSEs in an organism is influenced by 2 main factors: the similarity between the primary PrP sequence of the host (recipient) and the donor (inoculum), and the prion strain (*2*). Together, those factors define the concept of the transmission barrier. Sheep and goats share the same PrP primary sequence, although polymorphisms differ between the animals. In sheep, high susceptibility to classical scrapie is associated with the V_136_R_154_Q_171_ and A_136_R_154_Q_171_ alleles, whereas the A_136_R_154_R_171_ genotype is linked to resistance (*3*–*8*). To control and decrease classical scrapie in sheep, European Union member states have established breeding programs on the basis of the selection of the resistant A_136_R_154_R_171_ allele, although the variant does not confer resistance against the atypical/Nor98 scrapie strain (*9*). In goats, some polymorphisms, such as I_142_M (*10*–*13*) and N_146_S (*14*), have been associated with resistance to scrapie infection.

The most promising results of studies were in regard to goat-resistant polymorphisms for the goat Q_222_K polymorphism. The lysine allele (K_222_) was first reported to confer resistance in Italy (*15*,*16*), and similar results were later found in France (*10*) and Greece (*17*,*18*). Cell-free conversion assays also indicated that K_222_ provides protection against the ME7 scrapie strain (*19*). Experimental studies in goats found that heterozygous Q/K_222_ and homozygous K_222_ goats either showed resistance to classical scrapie or exhibited clear delays in incubation times after intracerebral or oral inoculation (*20*–*23*) and reduced contribution of K_222_ to proteinase K–resistant PrP (PrP^res^) formation in Q/K_222_ heterozygous goats infected with scrapie (*24*). In addition, Q/K_222_ heterozygous goats were found to harbor a relative abundance of the natural α-cleaved PrP^C^ fragment C1, which has also been detected in classical scrapie-resistant R_171_ sheep (*25*). Furthermore, Q/K_222_ heterozygous goats inoculated with goat BSE showed neither evidence of clinical prion disease nor PrP^Sc^ accumulation in the brain or peripheral tissues (*26*,*27*), but low infectivity was detected after long postinoculation times (*26*). Finally, 1 goat harboring the K_222_-PrP^C^ variant tested positive for atypical/Nor98 scrapie, indicating that the genotype may still be susceptible to this scrapie strain (*28*). All those results were replicated using a hemizygous transgenic mouse line expressing the K_222_-PrP^C^ allele, which was found to be resistant to several classical scrapie isolates and cattle BSE, while susceptible to goat or sheep BSE and atypical scrapie (*29*,*30*).

We conducted our study on the transgenic homozygous mouse line, along with its control counterpart harboring the wild-type glutamine allele (Q_222_). We intracranially inoculated the mice with several isolates representative of different categories of classical scrapie strains to test whether animals still remained uninfected, as previously reported (*29*), or if they mimicked the results found in homozygous goats (*22*).

## Methods

### Ethics Considerations

We performed animal experiments in strict accordance with the recommendations included in the guidelines of European Community Council 2010/63/UE and made all efforts to minimize animal suffering. The Committee on the Ethics of Animal Experiments of the Instituto Nacional de Investigación y Tecnología Agraria y Alimentaria and the General Directorate of the Madrid Community Government approved the study (permit nos. CEEA 2011–050, PROEX 263/15).

### Prion Transmission Studies

We intracranially inoculated 20 μL of 10% (wt/vol) brain homogenate from previously characterized classical scrapie isolates (Table 1) into the right parietal lobe of 5–7 transgenic mice (6–7 weeks old), which expressed either the wild-type goat PrP^C^ (Q_222_-Tg501) or the K_222_-PrP^C^ variant (K_222_-Tg516) (*29*,*30*) in homozygosity. PrP^C^ expression levels of both mice lines were 2- to 4-fold the physiologic levels found in goat brain (*29*). We used a 25-gauge disposable hypodermic needle to inoculate animals while they were anesthetized with isoflurane.

**Table 1:** Isolates used in study of classical scrapie prions in homozygous K_222_ transgenic mice*

Category	Isolate	Species	Origin	Goat PrP genotype†	Description	Supplier
I	198/9	Sheep	Italy	wt; S_240_S	Classical scrapie isolate from a naturally infected sheep	ISS
II	S2	Goat	Spain	wt; S_240_P	Classical scrapie isolate from a naturally infected goat	UNIZAR
	CP060146 (*22*)	Goat	France	wt	Classical scrapie isolate from an experimentally infected goat	ENVT
	CP060146/K_222_ (*22*)	Goat	France	K_222_	Classical scrapie isolate from an experimentally infected goat	ENVT
II + III	UKA2	Goat	United Kingdom	wt; S_240_P	Classical scrapie isolate from a naturally infected goat	APHA
	F14	Goat	France	wt; I_142_M, S_240_P	Classical scrapie isolate from a naturally infected goat	INRA
IV	F10	Goat	France	wt; S_240_P	Classical scrapie isolate from a naturally infected goat	INRA
	C1	Goat	Cyprus	wt	Classical scrapie isolate from a naturally infected goat	VS
Negative control	Healthy goat brain	Goat	France	wt	Brain from a noninfected goat	INRA

*Isolates were classified as previously described (*31*) on the basis of prion biochemical features when transmitted in transgenic mice expressing the bovine PrP (Bo-Tg110) and biologic features transmitted in mice expressing the ovine PrP (Q_222_-Tg501). APHA, Animal and Plant Health Agency, Surrey, United Kingdom; ENVT, École Nationale Vétérinaire deToulouse, Toulouse, France; INRA, French National Institute for Agricultural Research, Nouzilly, France; ISS, Istituto Superiore di Sanitá Animal, Rome, Italy; UNIZAR, Universidad de Zaragoza, Spain; VS, Veterinary Services, Nicosia, Cyprus; wt, wild-type. †The wt goat prion protein genotype is A_136_R_154_P_240_/A_136_R_154_P_240_. S240S, S240P and I142M refer to polymorphisms at specific codons of the PRNP gene.

After inoculation, we monitored mice daily and assessed their neurologic status twice a week. We euthanized animals when the progression of prion disease was evident, at the end of their lifespan (around 650 days postinoculation), or at previously established endpoints as part of a kinetic study. We harvested mouse brains and sliced them sagittally. We fixed half of each brain in 10% buffered formalin for histopathologic analysis and homogenized the remaining portion as 10% (wt/vol) in 5% glucose to detect PrP^res^ by Western blot.

We calculated survival time as the mean number days postinoculation for all mice that tested positive for PrP^res^ in the brain, with the SD included. We expressed attack rate as the proportion of PrP^res^-positive mice among all the inoculated mice.

### Western Blotting

We homogenized mouse brain tissue in 5% glucose solution in distilled water using grinding tubes (Bio-Rad Laboratories, https://www.bio-rad.com) and adjusted to 10% (wt/vol) using a TeSeE Precess 48TM homogenizer (Bio-Rad) according to the manufacturer’s instructions. We determined PrP^res^ presence in transgenic mouse brains by Western blot analysis of 10–100 µL of 10% (wt/vol) brain homogenate, as previously described (*32*). We incubated membranes with the Sha31 monoclonal antibody (mAb) (*33*), which recognizes the _148_YEDRYYRE_155_ epitope of the goat PrP sequence. We detected immunocomplexes with horseradish peroxidase-conjugated mouse IgG (GE HealthCare, https://www.gehealthcare.com) after 1 hour of incubation. We visualized immunoreactivity by chemiluminescence with ECL Select (GE HealthCare). We captured images using ChemiDoc XRS + System (Bio-Rad) and processed them using Image Lab 5.2.1 software (Bio-Rad).

### Histologic Analysis

To analyze brain tissue, we trimmed and dehydrated formalin-fixed brains, embedded them in paraffin wax, and cut 4-μm slices. We dewaxed and rehydrated the specimens by standard procedures. We established the vacuolar lesion profile of the brains in accordance with published standard methods and semiquantitatively scored vacuolation on a scale of 0–5 in different brain areas (*34*,*35*).

For immunohistochemical (IHC) demonstration of PrP^Sc^ accumulation, tissue sections underwent antigen retrieval and hydrogen peroxide quenching as previously described (*36*). We incubated the sections with 2A11 mAb (*37*), which recognizes the _163_QVYYRPVDQ_171_ epitope of the goat PrP sequence. Subsequently, we subjected the sections to antigen retrieval and inactivation of endogenous peroxidase activity before incubating them with the 2A11 mAb. We used a commercial immunoperoxidase technique (VECTASTAIN Elite ABC Kit; Vector Laboratories, https://vectorlabs.com), according to the manufacturer’s instructions. Finally, we counterstained the sections with Mayer’s hematoxylin. We used the Sha31 mAb (*33*) for paraffin-embedded tissue blotting, as previously described (*38*,*39*).

## Results

### Homozygous K_222_-Tg516 Mice and Resistance to Classical Scrapie PrP^Sc^

**Figure 1:**
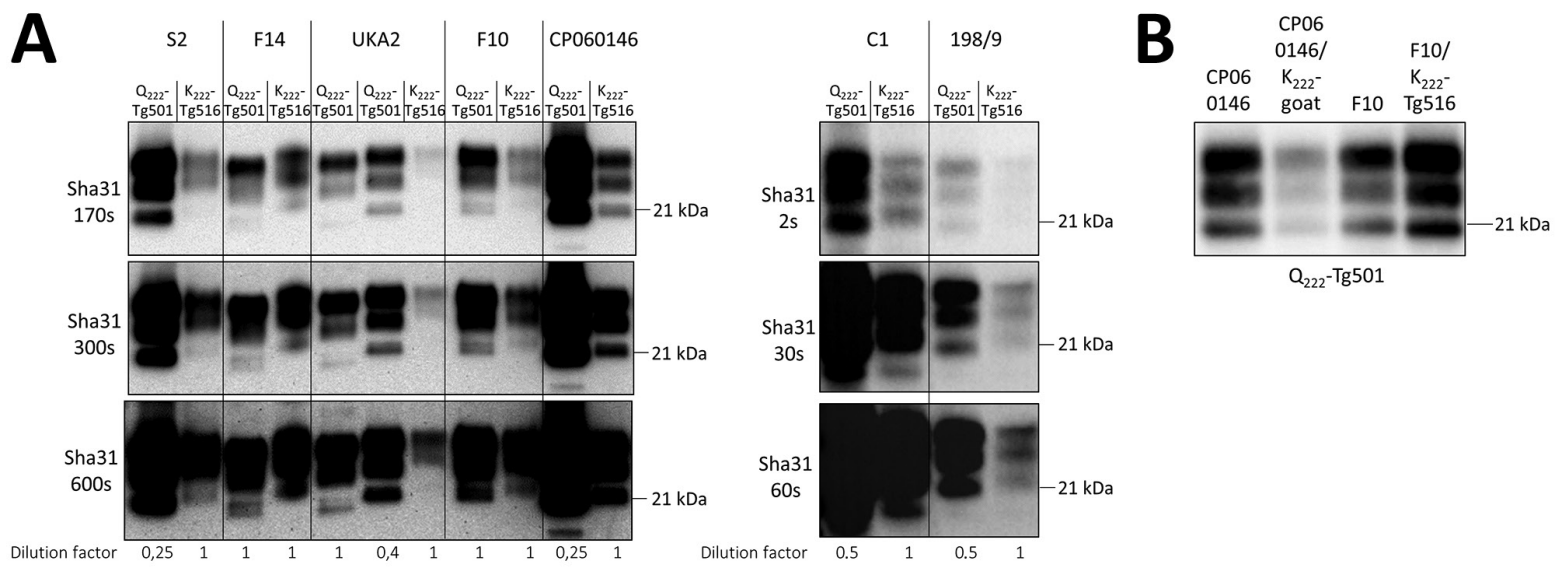
Proteinase K–resistant PrP (PrP^res^) accumulation in brains of K_222_-Tg516 and Q_222_-Tg501 homozygous mice in study of propagation of classical scrapie prions. A) Comparison of the biochemical profile of brain PrP^res^ from classical scrapie isolates in K_222_-Tg516 mice with that in Q_222_-Tg501 mice using Sha31 monoclonal antibody. Exposure time and dilution factor are specified. B) Comparison of the biochemical profile of brain PrP^res^ of CP060146 and F10 isolates of classical scrapie, before (left) and after (right) adaptation to the K_222_-PrP^C^ context, in Q_222_-Tg501 mice, using the Sha31 monoclonal antibody. Molecular weight markers are indicated on the right side of each band.

We intracranially inoculated homozygous K_222_-Tg516 with classical scrapie isolates (Table 1) previously characterized as representative of different prion strains circulating in Europe (*31**, **40*). Although all mice expressing the wild-type goat PrP (Q_222_-Tg501) developed recognizable prion disease, K_222_-Tg516 mice reached the end of their lifespan without showing clinical signs indicative of prion disease (Table 2). After second passage, survival times were still prolonged, even reaching the end of the mice’s lifespan again (Table 2). However, in both first and second passages, Western blot analysis showed the presence of PrP^res^ in the brains of K_222_-Tg516 animals inoculated with the different classical scrapie isolates (Figure 1, panel A). For the 198/9 and S2 isolate, the percentage of PrP^res^-positive animals in the first passage was not 100% of the inoculated animals (Tables 2, 3). At least for the S2 isolate, 100% of the inoculated mice were PrP^res^-positive by the completion of the second passage (Tables 2, 3). Comparison between the PrP^res^ signature of the original inoculum and the PrP^res^ obtained molecular mass for the nonglycosylated band, depending on the individual (Figure 1, panel A). In addition, brain PrP^res^ accumulation in K_222_-Tg516 mice was remarkably reduced compared with that in Q_222_-Tg501 mice for most of the inoculated isolates, with the exception of F14 and F10 (Figure 1, panel A).

**Table 2:** Transmission of classical scrapie isolates to Q_222_-Tg501 homozygous mice and survival of mice in study of classical scrapie prions in homozygous K_222_ transgenic mice*

Category	Isolate	1st passage			2nd passage	
		Mean survival time ± SD, d	No. diseased and PrP^res^-positive/no. inoculated		Mean survival time ± SD, d	No. diseased and PrP^res^-positive/no. inoculated
I	198/9	592 ± 13	6/6		536 ± 46	5/5
II	S2	228 ± 15	6/6		233 ± 4	6/6
	CP060146	379 ± 31	5/5		ND	NA
	CP060146/K_222_ goat	415 ± 40	6/6		ND	NA
II + III	UKA2	245 ± 36	5/5		252 ± 8	6/6
	F14	526 ± 46	4/4		241 ± 22	4/4
IV	F10	449 ± 19	5/5		372 ± 14	6/6
	F10/K_222_-Tg516	495 ± 26	3/3		ND	NA
	C1	483 ± 15	4/4		301 ± 10	4/4
Negative control	Healthy goat brain	>650	0/6†		>650	0/6†

*NA, not available; ND, not done; PrP^res^, proteinase K–resistant PrP. †Animals were found dead or were euthanized at the end of their lifespan without showing clinical signs of classical scrapie.

**Table 3:** Transmission of classical scrapie isolates to K_222_-Tg516 homozygous mice in study of classical scrapie prions in homozygous K_222_ transgenic mice*

Category	Isolate	1st passage			2nd passage		
		Mean survival time ± SD, d	No. diseased and PrP^res^-positive/no. inoculated		Mean survival time ± SD, d	No. diseased and PrP^res^-positive/no. inoculated	
I	198/9	>650	1/6†		ND	NA	
II	S2	>650	3/4†		>650	7/7†	
	CP060146	>650	5/5†		>650	5/5†	
	CP060146/K_222_ goat	>650	4/4†		>650	6/6†	
II + III	UKA2	>650	4/4†		>650	5/5†	
	F14	>650	4/4†		>650	5/5†	
IV	F10	>650	6/6†		>650	5/5†	
	F10/K_222_-Tg516	>650	5/5†		>650	6/6†	
	C1	>650	7/7†		ND	NA	
Negative control	Healthy goat brain	>650	0/6†		>650	0/6†	

*NA, not available; ND, not done; PrP^res^, proteinase K–resistant PrP. †Animals were found dead or euthanized at the end of their lifespan without showing clinical signs of classical scrapie.

**Figure 2:**
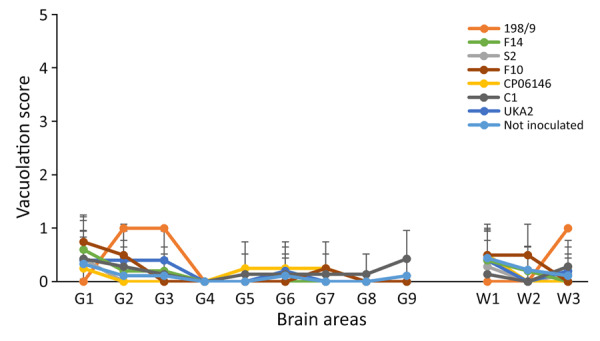
Histologic analysis of brain tissue from K_222_-Tg516 homozygous mice inoculated with classical scrapie in study of propagation of classical scrapie prions. Comparative analysis shows the vacuolar lesion profile in homozygous K_222_-Tg516 mice inoculated with different scrapie isolates compared with noninoculated mice. G, gray matter; W, white matter.

**Figure 3:**
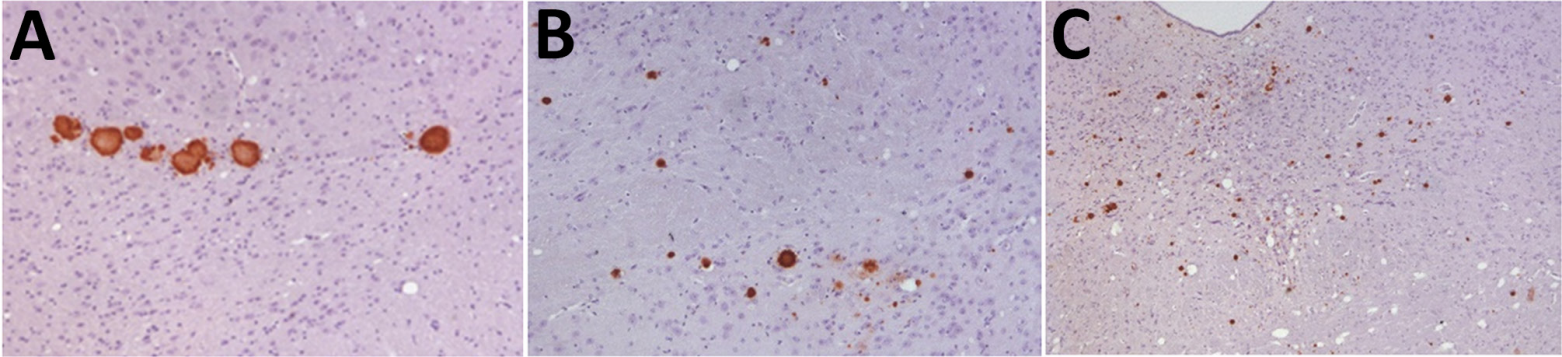
Immunohistochemistry results of brain tissue in study of propagation of classical scrapie prions. Images are of tissue specimens from K_222_-Tg516 mice inoculated with F10 goat scrapie isolate at second passage. Results are visualized using the Sha31 monoclonal antibody. A) Thalamus specimen. B) Hippocampus specimen. C) Midbrain specimen. Original magnification ×40.

**Figure 4:**
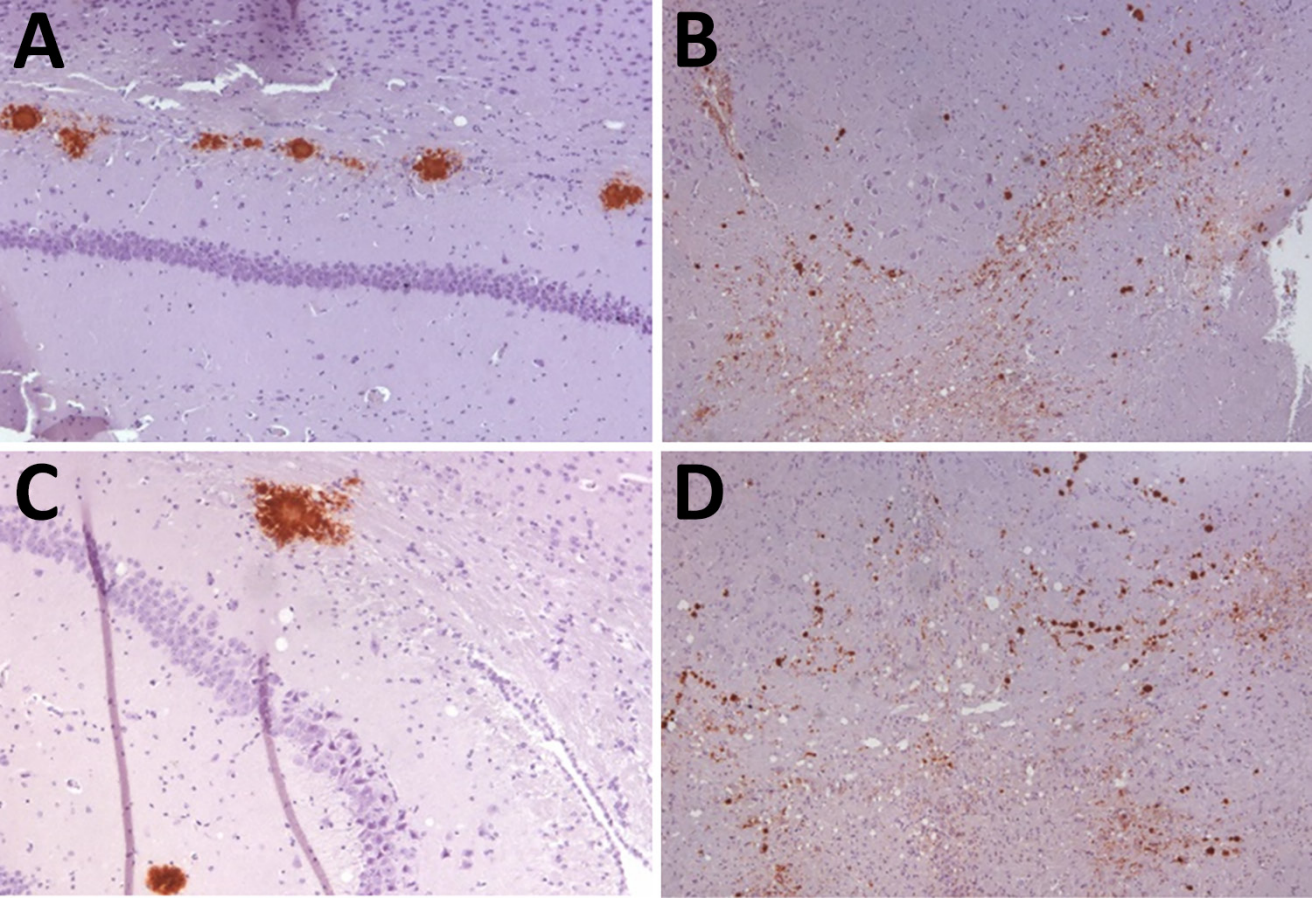
Immunohistochemistry results of brain tissues in study of propagation of classical scrapie prions. Images are of tissue specimens from K_222_-Tg516 mice inoculated with CP060146/K_222_ goat isolate. Results are visualized using the Sha31 monoclonal antibody. A) Hippocampus specimen tested at first passage. B) Midbrain specimen tested at first passage. C) Hippocampus specimen tested at second passage. D) Midbrain specimen tested at second passage. Original magnification ×40.

**Figure 5:**
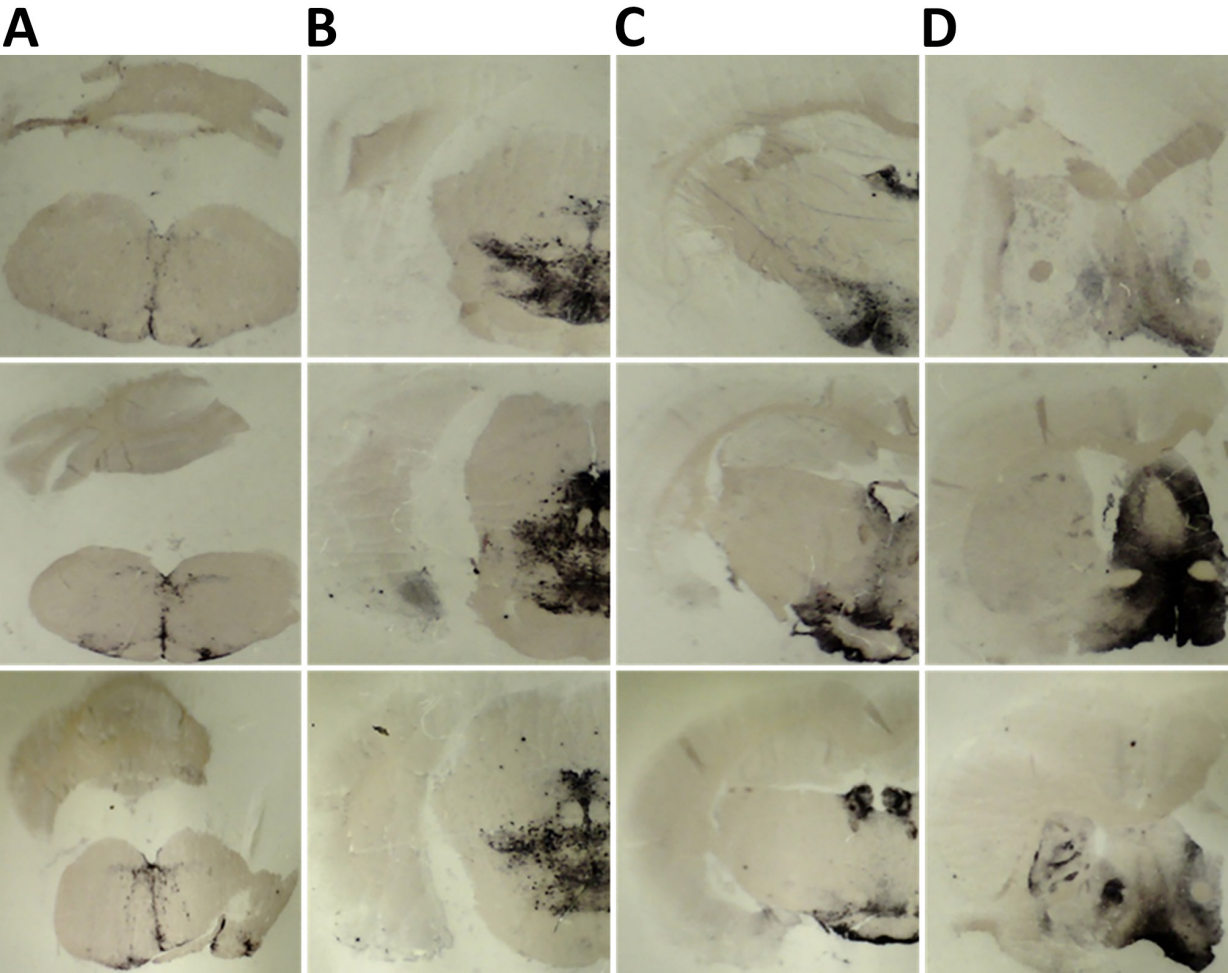
Paraffin-embedded tissue blotting results of brain tissues in study of propagation of classical scrapie prions. Images are of brain specimens from 3 distinct K_222_-Tg516 mice inoculated with CP060146/K_222_ goat isolate. Results are visualized with the Sha31 monoclonal antibody. A) Cerebellum specimens. B) Thalamus specimens. C) Hippocampus specimens. D) Cerebral cortex specimens. Proteinase K–resistant prion protein is visible as dark staining in similar brain regions in the 3 mice. Original magnification ×20.

K_222_-Tg516 PrP^res^-positive animals exhibited only a few vacuolations that were difficult to distinguish from those resulting from the physiologic aging process (Figure 2). Immunohistochemistry of K_222_-Tg516 mice inoculated with CP060146/K_222_ goat and F10/K_222_-Tg516 inocula revealed only a few large and focalized PrP^Sc^ plaques and lacked any other type of deposits affecting neurons or microglia cells (Figures 3, 4). Those PrP^Sc^ deposits were restricted to the mesencephalon, thalamus, and hypothalamus areas (Figures 3, 4). We detected no deposits for the remaining inoculations (data not shown). Consistent with our findings, paraffin-embedded tissue blotting showed clear PrP^res^ deposition only in K_222_-Tg516 mice inoculated with CP060146/K_222_ goat (Figure 5) and F10/K_222_-Tg516 inocula (data not shown), with deposition to the exact same brain areas affected by IHC (Figures 3, 4). We detected no deposits for the remaining inoculations (data not shown).

### Proteinase K Studies in K_222_-Tg516 Mice

**Figure 6:**
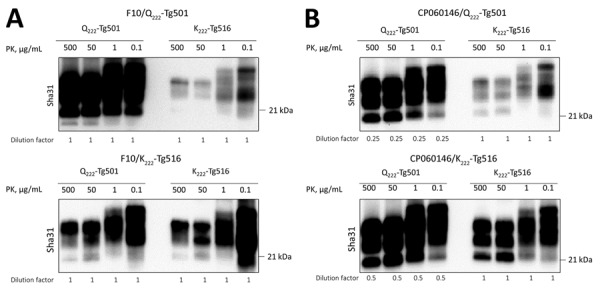
Proteinase K digestion studies conducted as part of study of propagation of classical scrapie prions. K_222_-Tg516 and Q_222_-Tg501 homozygous mice were inoculated with classical scrapie. A) Proteinase K–resistant prion protein (PrP^res^) sensitivity in the brains of Q_222_-Tg501 and K_222_-Tg516 mice initially inoculated with F10 scrapie isolate and subsequently reinoculated into both the original model and its counterpart. B) PrP^res^ sensitivity in the brains of Q_222_-Tg501 and K_222_-Tg516 mice initially inoculated with CP060146 scrapie isolate and subsequently reinoculated into both the original model and its counterpart. In both cases, proteinase K concentrations of 500, 50, 1 and 0.1 µg/mL were tested. Western blot visualizations were done using the Sha31 monoclonal antibody. Molecular weight markers are indicated on the right side of each band.

The differential brain PrP^res^ accumulation observed between K_222_-Tg516 and Q_222_-Tg501 mice (Figure 1, panel A) can be attributed to 2 alternative hypotheses. There could be a genuine reduction in PrP^res^ accumulation for these classical scrapie isolates in K_222_-Tg516 mice. Alternatively, the produced PrP^res^ might be more susceptible to proteinase K treatment, resulting in a weaker Western-blotting signal. To distinguish between those 2 possibilities, we performed proteinase K resistance analyses using different enzyme concentrations in both Q_222_-Tg501 and K_222_-Tg516 mice inoculated with F10 (which exhibited similar PrP^res^ accumulation between K_222_-Tg516 and Q_222_-Tg501 mice) and CP060146 (which showed reduced PrP^res^ accumulation in K_222_-Tg516 mice compared with Q_222_-Tg501 mice) isolates. In all cases, proteinase K consistently acted at a concentration of 50 µg/mL (which falls within the normal proteinase K concentration range for routine Western blotting); we observed the same pattern and signal intensity at a concentration of 500 µg/mL (Figure 6). However, protease action did not achieve proper PrP^res^ resolution at concentrations of 1 µg/mL and 0.1 µg/mL (Figure 6). Those results suggest that both isolates, when replicating in either Q_222_-PrP^C^ or K_222_-PrP^C^ contexts, retain the same proteinase K sensitivity. Thus, the differences in Western blotting signals detected previously (Figure 1, panel A) truly account for reduced brain PrP^res^ accumulation in K_222_-Tg516 mice.

### Transmission in K_222_-Tg516 Mice and Host-Induced Reversible Strain Adaptations

After the second passage in K_222_-Tg516 mice or adaptation in a K_222_ homozygous goat, F10 and CP060146 isolates were transmitted back into Q_222_-Tg501 mice (Table 3). The purpose of those inoculations was to determine whether replication in the K_222_ context resulted in host-induced reversible adaptations of the strain, including changes in prion strain characteristics such as biologic properties (mean survival time and proportion of PrP^res^-positive animals) and biochemical properties (brain PrP^res^ accumulation and PrP^res^ glycosylation pattern). In both cases, the survival times were comparable to those observed for the primary transmission of the same inocula in Q222-Tg501 mice. Specifically, survival time was 449 + 19 days (5/5) for the original F10 inoculum from a wild-type goat versus 495 + 26 days (3/3) after adaptation in K_222_-Tg516 mice and 379 ± 31 days (5/5) for the original CP060146 inoculum from a wild-type goat versus 415 + 40 days (6/6) after adaptation in a K_222_ goat (Table 2). The PrP^res^ signatures obtained were identical to those observed after the primary transmission of these isolates in Q_222_-Tg501 mice (Figure 1, panel B).

### Differences between K_222_ and Q_222_ PrP^res^ Formation Kinetics 

**Figure 7:**
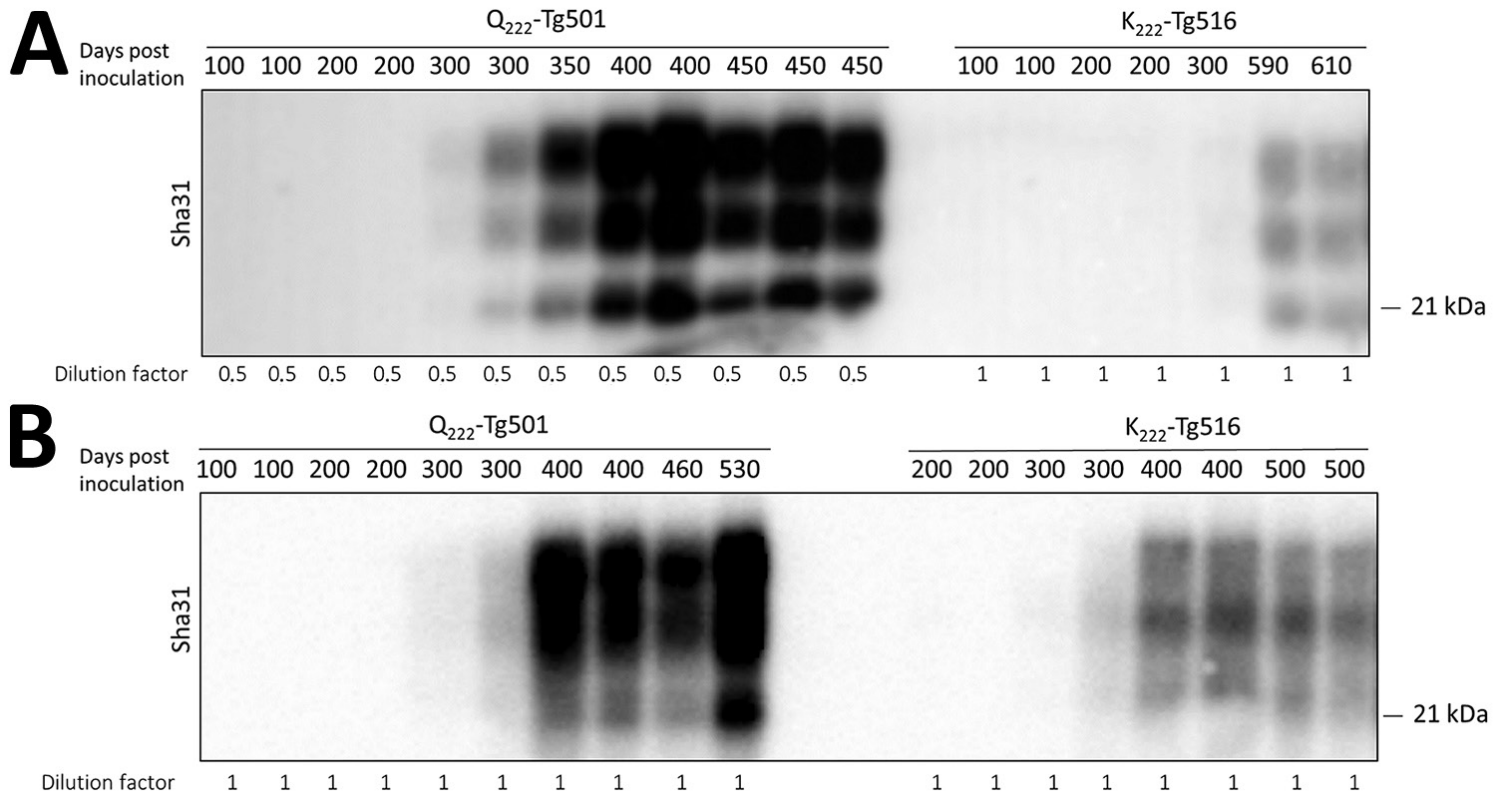
Kinetic studies of proteinase K–resistant prion protein (PrP^res^) detection in K_222_-Tg516 and Q_222_-Tg501 homozygous mice inoculated with classical scrapie in study of propagation of classical scrapie prions. Brain PrP^res^ from mice euthanized at various time points postinoculation were analyzed by Western blotting and visualized using the Sha31 monoclonal antibody. A) Q_222_-Tg501 and K_222_-Tg516 mice inoculated with the CP060146 classical scrapie isolate adapted to the K_222_ cellular prion protein (PrP^C^) context (CP060146/K_222_-goat). B) Q_222_-Tg501 and K_222_-Tg516 mice inoculated with the F10 classical scrapie isolate adapted to the K_222_-PrP^C^ context (F10/K_222_-Tg516). Molecular weight markers are indicated on the right side of each band.

Once we confirmed the lower brain PrP^res^ accumulation in K_222_-Tg516 mice compared with the Q_222_-Tg501 control counterparts, we conducted kinetic studies on PrP^res^ formation in both transgenic lines using the goat isolates F10 and CP060146, which had been previously adapted for propagation in a K_222_-PrP^C^ context. Of interest, both K_222_ and Q_222_-PrP^res^ appeared at equal levels by 300 days postinoculation (Figure 7). However, Q_222_-PrP^res^ accumulation continued to increase steadily until the time of death, whereas K_222_-PrP^res^ remained at low levels throughout the lifespan of the mice (Figure 7).

## Discussion

Previous studies conducted in heterozygous Q/K_222_ and homozygous K_222_ goats (*20*–*23*), as well as in hemizygous K_222_-Tg516 mice (*29*), have highlighted the Q_222_K polymorphism as one of the most promising candidates for reducing prion disease transmission in goats. Although the K_222_ allele has been consistently reported in certain countries in Europe, such as Italy (*15*,*16*), France (*10*), and Greece (*17*,*42*), in other countries, such as the United Kingdom, the polymorphism has been reported as infrequent (*43*). However, once the supposed protective effect against prion diseases is confirmed, the frequency of the K_222_ allele could increase across different countries through selective breeding programs.

Transgenic mice expressing K_222_-PrP^C^ in homozygosity emerge as the optimal tool for definitively testing the susceptibility or resistance that allele confers to prions. Our model enables the testing of multiple prion strains more rapidly and cost-effectively than the model using goats. In our study, classical scrapie isolates representing different classical scrapie strains circulating within Europe (*40*–*42*) were selected and used to challenge homozygous K_222_-Tg516 mice.

Once the expression level is increased, homozygous K_222_-Tg516 mice become susceptible to all tested classical scrapie isolates (Table 3). The K_222_-PrP^C^ variant is capable of sustaining PrP^Sc^ replication even in the absence of the Q_222_-PrP^C^ variant, which was identified as responsible for most accumulated brain PrP^res^ in Q_222_K heterozygous goats (*24*). Furthermore, K_222_-Tg516 mice exhibit consistently lower brain PrP^res^ accumulation than Q_222_-Tg501 mice (Figure 1). The explanation that K_222_-PrP^res^ is more sensitive to proteinase K treatment and so reduced detection of brain PrP^res^ accumulation has been ruled out (Figure 6). Therefore, we recommend careful analysis of the general features and behavior of classical scrapie K_222_-PrP^res^.

K_222_-Tg516 mice inoculated with classical scrapie did not develop typical prion pathology and showed no clinical signs of prion disease, which suggests that classical scrapie K_222_-PrP^res^ might not be toxic or might not induce the signaling pathways leading to neuronal death. Those conclusions are not only caused by insufficient time for the onset of neuronal death pathways within the animal lifespan; second passages in K_222_-Tg516 yielded identical results to the first ones. However, we noted that the lower brain PrP^res^ accumulation in K_222_-Tg516 animals could lead to a misinterpretation of those results. The reduced accumulation might reflect insufficient replication within the animal’s lifespan, possibly caused by consistently low replication rates, as suggested by our kinetic experiments, or by more efficient clearance of PrP^res^ aggregates. Those factors could explain why transmission does not necessarily result in prion disease, highlighting a dissociation between infectivity and toxicity of classical scrapie K_222_-PrP^res^.

All circulating prion strains must be considered in the design of breeding selection programs. Programs aimed at controlling and reducing classical scrapie in sheep, implemented by EU member states, have identified sheep herds that are more susceptible to atypical/Nor98 scrapie (*44*). In our study, K_222_-Tg516 mice died without exhibiting overt clinical signs after inoculation with different classical scrapie isolates; we found that PrP^res^ accumulated in their brains (Table 1). Of note, K_222_-derived PrP^res^ retained infectivity when transmitted back to Q_222_-Tg501 mice, recovering the strain characteristics observed in the original inocula. Our findings suggest that, under the experimental conditions we established, the K_222_ allele does not confer full resistance to classical scrapie agents.

Of interest, the reversibility of strain features observed upon reinoculation of K_222_-derived PrP^res^ into Q_222_-Tg501 mice is reminiscent of the phenomenon of nonadaptive prion amplification as described previously (*45*). In that model, PrP^Sc^ can replicate transiently in a nonpermissive host without inducing a permanent adaptation of the strain. Our data are consistent with that concept; the classical scrapie agents replicated in K_222_-Tg516 mice but reverted to their original biochemical and biologic properties upon passage back into a permissive Q_222_ context. That interpretation reinforces the view that the K_222_ allele may enable subclinical or low-efficiency replication of classical scrapie agents without supporting stable strain selection or adaptation.

It is important to note that the use of transgenic models with PrP overexpression may enhance prion replication efficiency, potentially uncovering low-level or subclinical conversion events that might not occur under physiologic PrP expression in goats. In addition, all animals were inoculated intracerebrally; that route does not mimic natural exposure and bypasses key peripheral barriers such as the gut and associated lymphoid tissues, which play a critical role in determining prion susceptibility and pathogenesis under field conditions. Therefore, although our results highlight the potential for silent propagation of classical scrapie strains in the context of the K_222_ variant, extrapolation to the natural host should be made with caution.

Interest has grown for in-depth characterization of the strains of Q/K_222_ heterozygous goats affected with scrapie, which are abundant in various regions of Greece. The interest lies in determining whether prions propagated under the K_222_ allele can act as potential silent carriers of the disease, as shown in previous studies. Furthermore, understanding whether the presence of the K_222_ allele induces a change in the biologic properties of the strains and their potential transmission to other animal species is crucial.

Overall, our results underscore the need for further in vivo studies using physiologically relevant models or natural hosts to fully evaluate the protective efficacy of the K_222_ allele. Until such evidence becomes available, the inclusion of the K_222_ polymorphism in breeding selection programs should be critically considered, especially in regions where classical scrapie strains with known zoonotic potential remain present. Furthermore, experiments conducted in classical BSE-inoculated Q/K_222_ heterozygous goats have shown at least low infectivity in goat tissues after long postinoculation periods (*26*), whereas heterozygous K_222_-Tg516 mice were already fully susceptible to goat BSE (*29*). In addition, at least 1 Q/K_222_ heterozygous goat tested positive for atypical/Nor98 scrapie (*28*), and homozygous K_222_-Tg516 mice were found to be completely susceptible to atypical/Nor98 scrapie (*30*). Taken together, those data suggest that the protective effect of the Q_222_K polymorphism may be limited, and its use in breeding programs should be carefully evaluated.

## References

[1] Prusiner SB. Molecular biology of prion diseases. Science. 1991;252:1515–22. 10.1126/science.1675487 1675487

[2] Marín-Moreno A, Fernández-Borges N, Espinosa JC, Andréoletti O, Torres JM. Transmission and replication of prions. Prog Mol Biol Transl Sci. 2017;150:181–201. 10.1016/bs.pmbts.2017.06.014 28838661

[3] Baylis M, Chihota C, Stevenson E, Goldmann W, Smith A, Sivam K, et al. Risk of scrapie in British sheep of different prion protein genotype. J Gen Virol. 2004;85:2735–40. 10.1099/vir.0.79876-0 15302967

[4] Houston F, Goldmann W, Foster J, González L, Jeffrey M, Hunter N. Comparative susceptibility of sheep of different origins, breeds and PRNP genotypes to challenge with bovine spongiform encephalopathy and scrapie. PLoS One. 2015;10:e0143251. 10.1371/journal.pone.0143251 26587837 PMC4654545

[5] Belt PB, Muileman IH, Schreuder BE, Bos-de Ruijter J, Gielkens AL, Smits MA. Identification of five allelic variants of the sheep PrP gene and their association with natural scrapie. J Gen Virol. 1995;76:509–17. 10.1099/0022-1317-76-3-509 7897344

[6] Bossers A, Schreuder BE, Muileman IH, Belt PB, Smits MA. PrP genotype contributes to determining survival times of sheep with natural scrapie. J Gen Virol. 1996;77:2669–73. 10.1099/0022-1317-77-10-2669 8887505

[7] Hunter N, Foster JD, Goldmann W, Stear MJ, Hope J, Bostock C. Natural scrapie in a closed flock of Cheviot sheep occurs only in specific PrP genotypes. Arch Virol. 1996;141:809–24. 10.1007/BF01718157 8678828

[8] Hunter N, Moore L, Hosie BD, Dingwall WS, Greig A. Association between natural scrapie and PrP genotype in a flock of Suffolk sheep in Scotland. Vet Rec. 1997;140:59–63. 10.1136/vr.140.3.59 9023905

[9] Benestad SL, Arsac JN, Goldmann W, Nöremark M. Atypical/Nor98 scrapie: properties of the agent, genetics, and epidemiology. Vet Res. 2008;39:19. 10.1051/vetres:2007056 18187032

[10] Barillet F, Mariat D, Amigues Y, Faugeras R, Caillat H, Moazami-Goudarzi K, et al. Identification of seven haplotypes of the caprine *PrP* gene at codons 127, 142, 154, 211, 222 and 240 in French Alpine and Saanen breeds and their association with classical scrapie. J Gen Virol. 2009;90:769–76. 10.1099/vir.0.006114-0 19218225

[11] González L, Martin S, Hawkins SA, Goldmann W, Jeffrey M, Sisó S. Pathogenesis of natural goat scrapie: modulation by host PRNP genotype and effect of co-existent conditions. Vet Res. 2010;41:48. 10.1051/vetres/2010020 20374697 PMC2865875

[12] Goldmann W, Ryan K, Stewart P, Parnham D, Xicohtencatl R, Fernandez N, et al. Caprine prion gene polymorphisms are associated with decreased incidence of classical scrapie in goat herds in the United Kingdom. Vet Res. 2011;42:110. 10.1186/1297-9716-42-110 22040234 PMC3224758

[13] Goldmann W, Martin T, Foster J, Hughes S, Smith G, Hughes K, et al. Novel polymorphisms in the caprine *PrP* gene: a codon 142 mutation associated with scrapie incubation period. J Gen Virol. 1996;77:2885–91. 10.1099/0022-1317-77-11-2885 8922485

[14] Papasavva-Stylianou P, Windl O, Saunders G, Mavrikiou P, Toumazos P, Kakoyiannis C. *PrP* gene polymorphisms in Cyprus goats and their association with resistance or susceptibility to natural scrapie. Vet J. 2011;187:245–50. 10.1016/j.tvjl.2009.10.015 20093056

[15] Vaccari G, Bari MAD, Morelli L, Nonno R, Chiappini B, Antonucci G, et al. Identification of an allelic variant of the goat *PrP* gene associated with resistance to scrapie. J Gen Virol. 2006;87:1395–402. 10.1099/vir.0.81485-0 16603543

[16] Acutis PL, Bossers A, Priem J, Riina MV, Peletto S, Mazza M, et al. Identification of prion protein gene polymorphisms in goats from Italian scrapie outbreaks. J Gen Virol. 2006;87:1029–33. 10.1099/vir.0.81440-0 16528054

[17] Bouzalas IG, Dovas CI, Banos G, Papanastasopoulou M, Kritas S, Oevermann A, et al. Caprine PRNP polymorphisms at codons 171, 211, 222 and 240 in a Greek herd and their association with classical scrapie. J Gen Virol. 2010;91:1629–34. 10.1099/vir.0.017350-0 20107013

[18] Fragkiadaki EG, Vaccari G, Ekateriniadou LV, Agrimi U, Giadinis ND, Chiappini B, et al. PRNP genetic variability and molecular typing of natural goat scrapie isolates in a high number of infected flocks. Vet Res. 2011;42:104. 10.1186/1297-9716-42-104 21961834 PMC3190342

[19] Eiden M, Soto EO, Mettenleiter TC, Groschup MH. Effects of polymorphisms in ovine and caprine prion protein alleles on cell-free conversion. Vet Res. 2011;42:30. 10.1186/1297-9716-42-30 21324112 PMC3050705

[20] Acutis PL, Martucci F, D’Angelo A, Peletto S, Colussi S, Maurella C, et al. Resistance to classical scrapie in experimentally challenged goats carrying mutation K222 of the prion protein gene. Vet Res. 2012;43:8. 10.1186/1297-9716-43-8 22296670 PMC3296670

[21] White SN, Reynolds JO, Waldron DF, Schneider DA, O’Rourke KI. Extended scrapie incubation time in goats singly heterozygous for PRNP S146 or K222. Gene. 2012;501:49–51. 10.1016/j.gene.2012.03.068 22516690

[22] Lacroux C, Perrin-Chauvineau C, Corbière F, Aron N, Aguilar-Calvo P, Torres JM, et al. Genetic resistance to scrapie infection in experimentally challenged goats. J Virol. 2014;88:2406–13. 10.1128/JVI.02872-13 24284317 PMC3958109

[23] Cinar MU, Schneider DA, Waldron DF, O’Rourke KI, White SN. Goats singly heterozygous for PRNP S146 or K222 orally inoculated with classical scrapie at birth show no disease at ages well beyond 6 years. Vet J. 2018;233:19–24. 10.1016/j.tvjl.2017.12.019 29486874

[24] Mazza M, Guglielmetti C, Pagano M, Sciuto S, Ingravalle F, Martucci F, et al. Lysine at position 222 of the goat prion protein inhibits the binding of monoclonal antibody F99/97.6.1. J Vet Diagn Invest. 2012;24:971–5. 10.1177/1040638712457352 22914824

[25] Madsen-Bouterse SA, Stewart P, Williamson H, Schneider DA, Goldmann W. Caprine PRNP polymorphisms N146S and Q222K are associated with proteolytic cleavage of PrP^C^. Genet Sel Evol. 2021;53:52. 10.1186/s12711-021-00646-x 34147084 PMC8214774

[26] Aguilar-Calvo P, Fast C, Tauscher K, Espinosa J-C, Groschup MH, Nadeem M, et al. Effect of Q211 and K222 PRNP polymorphic variants in the susceptibility of goats to oral infection with goat bovine spongiform encephalopathy. J Infect Dis. 2015;212:664–72. 10.1093/infdis/jiv112 25722297

[27] Fast C, Goldmann W, Berthon P, Tauscher K, Andréoletti O, Lantier I, et al. Protecting effect of PrP codons M142 and K222 in goats orally challenged with bovine spongiform encephalopathy prions. Vet Res. 2017;48:52. 10.1186/s13567-017-0455-0 28927447 PMC5606029

[28] Colussi S, Vaccari G, Maurella C, Bona C, Lorenzetti R, Troiano P, et al. Histidine at codon 154 of the prion protein gene is a risk factor for Nor98 scrapie in goats. J Gen Virol. 2008;89:3173–6. 10.1099/vir.0.2008/004150-0 19008408

[29] Aguilar-Calvo P, Espinosa JC, Pintado B, Gutiérrez-Adán A, Alamillo E, Miranda A, et al. Role of the goat K222-PrP(C) polymorphic variant in prion infection resistance. J Virol. 2014;88:2670–6. 10.1128/JVI.02074-13 24352451 PMC3958052

[30] Aguilar-Calvo P, Espinosa JC, Andréoletti O, González L, Orge L, Juste R, et al. Goat K_222_-PrP^C^ polymorphic variant does not provide resistance to atypical scrapie in transgenic mice. Vet Res. 2016;47:96. 10.1186/s13567-016-0380-7 27659200 PMC5034450

[31] Marín-Moreno A, Aguilar-Calvo P, Espinosa JC, Zamora-Ceballos M, Pitarch JL, González L, et al. Classical scrapie in small ruminants is caused by at least four different prion strains. Vet Res. 2021;52:57. 10.1186/s13567-021-00929-7 33858518 PMC8048364

[32] Padilla D, Béringue V, Espinosa JC, Andreoletti O, Jaumain E, Reine F, et al. Sheep and goat BSE propagate more efficiently than cattle BSE in human PrP transgenic mice. PLoS Pathog. 2011;7:e1001319. 10.1371/journal.ppat.1001319 21445238 PMC3060172

[33] Féraudet C, Morel N, Simon S, Volland H, Frobert Y, Créminon C, et al. Screening of 145 anti-PrP monoclonal antibodies for their capacity to inhibit PrPSc replication in infected cells. J Biol Chem. 2005;280:11247–58. 10.1074/jbc.M407006200 15618225

[34] Marín-Moreno A, Huor A, Espinosa JC, Douet JY, Aguilar-Calvo P, Aron N, et al. Radical change in zoonotic abilities of atypical BSE prion strains as evidenced by crossing of sheep species barrier in transgenic mice. Emerg Infect Dis. 2020;26:1130–9. 10.3201/eid2606.181790 32441630 PMC7258450

[35] Fraser H, Dickinson AG. The sequential development of the brain lesion of scrapie in three strains of mice. J Comp Pathol. 1968;78:301–11. 10.1016/0021-9975(68)90006-6 4970192

[36] González L, Martin S, Houston FE, Hunter N, Reid HW, Bellworthy SJ, et al. Phenotype of disease-associated PrP accumulation in the brain of bovine spongiform encephalopathy experimentally infected sheep. J Gen Virol. 2005;86:827–38. 10.1099/vir.0.80299-0 15722546

[37] Brun A, Castilla J, Ramírez MA, Prager K, Parra B, Salguero FJ, et al. Proteinase K enhanced immunoreactivity of the prion protein-specific monoclonal antibody 2A11. Neurosci Res. 2004;48:75–83. 10.1016/j.neures.2003.09.004 14687883

[38] Andréoletti O, Berthon P, Levavasseur E, Marc D, Lantier F, Monks E, et al. Phenotyping of protein-prion (PrPsc)-accumulating cells in lymphoid and neural tissues of naturally scrapie-affected sheep by double-labeling immunohistochemistry. J Histochem Cytochem. 2002;50:1357–70. 10.1177/002215540205001009 12364569

[39] Andréoletti O, Simon S, Lacroux C, Morel N, Tabouret G, Chabert A, et al. PrPSc accumulation in myocytes from sheep incubating natural scrapie. Nat Med. 2004;10:591–3. 10.1038/nm1055 15156203

[40] Langeveld JPM, Pirisinu L, Jacobs JG, Mazza M, Lantier I, Simon S, et al. Four types of scrapie in goats differentiated from each other and bovine spongiform encephalopathy by biochemical methods. Vet Res. 2019;50:97. 10.1186/s13567-019-0718-z 31767033 PMC6878695

[41] Nonno R, Marin-Moreno A, Carlos Espinosa J, Fast C, Van Keulen L, Spiropoulos J, et al. Characterization of goat prions demonstrates geographical variation of scrapie strains in Europe and reveals the composite nature of prion strains. Sci Rep. 2020;10:19. 10.1038/s41598-019-57005-6 31913327 PMC6949283

[42] Kanata E, Humphreys-Panagiotidis C, Giadinis ND, Papaioannou N, Arsenakis M, Sklaviadis T. Perspectives of a scrapie resistance breeding scheme targeting Q211, S146 and K222 caprine PRNP alleles in Greek goats. Vet Res. 2014;45:43. 10.1186/1297-9716-45-43 24717012 PMC4030296

[43] Goldmann W, Marier E, Stewart P, Konold T, Street S, Langeveld J, et al. Prion protein genotype survey confirms low frequency of scrapie-resistant K222 allele in British goat herds. Vet Rec. 2016;178:168. 10.1136/vr.103521 26755614 PMC4789823

[44] Fediaevsky A, Tongue SC, Nöremark M, Calavas D, Ru G, Hopp P. A descriptive study of the prevalence of atypical and classical scrapie in sheep in 20 European countries. BMC Vet Res. 2008;4:19. 10.1186/1746-6148-4-19 18544152 PMC2442063

[45] Bian J, Khaychuk V, Angers RC, Fernández-Borges N, Vidal E, Meyerett-Reid C, et al. Prion replication without host adaptation during interspecies transmissions. Proc Natl Acad Sci U S A. 2017;114:1141–6. 10.1073/pnas.1611891114 28096357 PMC5293081

